# Effect of non-pharmacological interventions on the COVID-19 epidemic in Saudi Arabia

**DOI:** 10.1017/S0950268821002612

**Published:** 2021-11-29

**Authors:** Naif I. AlJohani, Kipkoech Mutai

**Affiliations:** 1 Harvard T. H. Chan School of Public Health, Boston, MI, USA; 2 Consultant at King Faisal Specialist Hospital & Research Centre, Jeddah, Saudi Arabia; 3 Consultant at Stat-Simp Consulting, Eldoret, Kenya

**Keywords:** Epidemiology, modelling, public health

## Abstract

We quantified the potential impact of different social distancing and self-isolation scenarios on the coronavirus disease 2019 (COVID-19) pandemic trajectory in Saudi Arabia and compared the modelling results to the confirmed epidemic trajectory. Using the susceptible, exposed, infected, quarantined and self-isolated, requiring hospitalisation, recovered/immune individuals, fatalities model, we assessed the impact of a non-pharmacological interventions’ subset. An unmitigated scenario (baseline), mitigation scenarios (25% reduction in social contact/twofold increase in self-isolation) and enhanced mitigation scenarios (50% reduction in social contact/twofold increase in self-isolation) were assessed and compared to the actual epidemic trajectory. For the unmitigated scenario, mitigation scenarios, enhanced mitigation scenarios and actual observed epidemic, the peak daily incidence rates (per 10 000 population) were 77.00, 16.00, 9.00 and 1.14 on days 71, 54, 35 and 136, respectively. The peak fatality rates were 35.00, 13.00, 5.00 and 0.016 on days 150, 125, 60 and 155, respectively. The R0 was 1.15, 1.14, 1.22 and 2.50, respectively. Aggressive implementation of social distancing and self-isolation contributed to the downward trend of the disease. We recommend using extensive models that comprehensively consider the natural history of COVID-19, social and behavioural patterns, age-specific data, actual network topology and population to elucidate the epidemic's magnitude and trajectory.

## Introduction

**Fig. 1.**
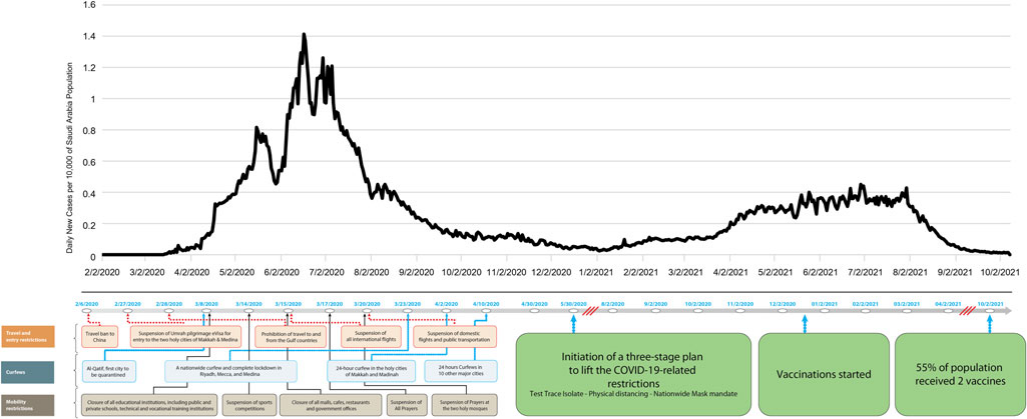
Non-pharmacological interventions employed by the Saudi Arabian government and their effects on the number of daily confirmed cases.

The first case of coronavirus disease 2019 (COVID-19) in Saudi Arabia was announced on 2 March 2020 [1]. By 8 May 2021, Saudi Arabia was the seventh most affected country in the Eastern Mediterranean region after Iran, Israel, Iraq, Jordan, United Arab Emirates and Lebanon. At the time, there were more than 424 000 cases but a comparatively low case fatality rate (7045 deaths, 1.87 deaths/10 000 population) [2]. On 25 February 2020, Saudi Arabia took early and aggressive preventive action to curb the spread of COVID-19 by prohibiting entry from and advising against travel to Italy and Japan and continued to progressively initiate additional strict non-pharmacological interventions (NPIs), including a variety of travel bans, curfews, aggressive mobility restrictions and increased testing [3]. Strict restrictions on mass gatherings were implemented, such as the curtailment of the Umrah (the annual pilgrimage to Mecca observed by Muslims), limitations on the number of people allowed for the Hajj pilgrimage and intermittent bans on prayers in mosques (Fig. 1) [4].

Mathematical models are used in infectious disease epidemiology to investigate and quantify the spread of the disease [5]. These models can be used for various reasons, such as when direct experimental studies investigating the spread of disease among humans may be unethical [6]. Mathematical models are also used to estimate the resources that will be required by a country's health system [7], determine the efficacy of various public health interventions in reducing the associated morbidity and mortality, and implement a timely and adequate response [5]. Stochastic models, using surveillance, temporal, clinical and demographic data, can help investigate transmission patterns of an infectious disease in heterogeneous populations [8]. During the COVID-19 epidemic, stochastic models were used in India to predict the impact of COVID-19 on health care [9], in Japan to assess the effectiveness of avoiding large gatherings or crowded areas [10], and in New York City to predict the impact of lifting restrictions on movement [11]. Outcome comparisons can be made based on the comparison of the observed results with the predicted results over time [12].

Giordano *et al*. used an eight-stage model of infection, termed SIDARTHE: susceptible (S), infected (I), diagnosed (D), ailing (A), recognised (R), threatened (T), healed (H) and extinct (E), to differentiate diagnosed from undiagnosed individuals who usually reside in the community and compared simulation results with real data on the COVID-19 epidemic in Italy. They found that social distancing delays the epidemic peak, whereas timely diagnosis reduces the infection peak and helps end the epidemic faster [13]. This study aimed to quantify the potential impact of NPIs (specifically different social distancing scenarios) on the trajectory of the COVID-19 pandemic in the Kingdom of Saudi Arabia and compare the modelling results to the actual trajectory of the epidemic in the country after approximately one year of the pandemic (Fig. 1).

## Methods

### The epidemic model

Mathematical modelling of infectious disease dynamics can be grouped into three broad categories, i.e. (i) statistical-based methods for epidemic surveillance (e.g. spatial models); (ii) mathematical/mechanistic state-space models (e.g. agent-based simulation); and (iii) empirical/machine learning-based models (e.g. web-based data mining) [14]. Throughout the COVID-19 pandemic, various mathematical models have been used for various purposes [14, 15]. Such models include mass action compartmental models (commonly referred to as susceptible (S), infective (I) and removed or recovered (R) [SIR] class of models), structural metapopulation models and agent-based network models [15]. Our model was an extension of an existing susceptible-infectious-recovered stochastic individual compartmental model within the EpiModel library in R (https://www.epimodel.org/), which was developed by researchers at the Rollins School of Public Health, Emory University [16].

In brief, Churches [17] developed this model as an extension of the EpiModel [16] to generate a susceptible-exposed-infectious-quarantined-hospitalised-recovered-fatal (SEIQHRF) stochastic individual compartmental model.

In the seven-compartment model, S represents susceptible populations who could potentially become infected, E represents exposed infected asymptomatic infectious individuals, I represents infected and infectious symptomatic individuals, Q represents quarantined but self-isolated infectious individuals, H represents individuals requiring hospitalisation including those who are actually hospitalised and those who would normally be hospitalised if the capacity were available, R represents recovered/immune individuals who are now immune from further infection, and F represents those who were infected and have died due to COVID-19 and not due to other causes [17].

The SEIQHRF model simulates the movement of a population across these seven compartments based on various transition rates and under different scenarios. Healthy people who are susceptible (S) may become exposed (E) to the virus and infected. Infectious asymptomatic individuals (I) and those who are infected and infectious (I) could move into one of the following four states:

- Clear their infection and return to the susceptible compartment (S)
- Self-isolate (Q)
  - and either clear infection and return to the susceptible compartment (S), require hospitalisation (H) or recover (R)
- Require hospitalisation (H)
  - and either recover (R) or die (F)
- Recover (R)

**Fig. 2.**
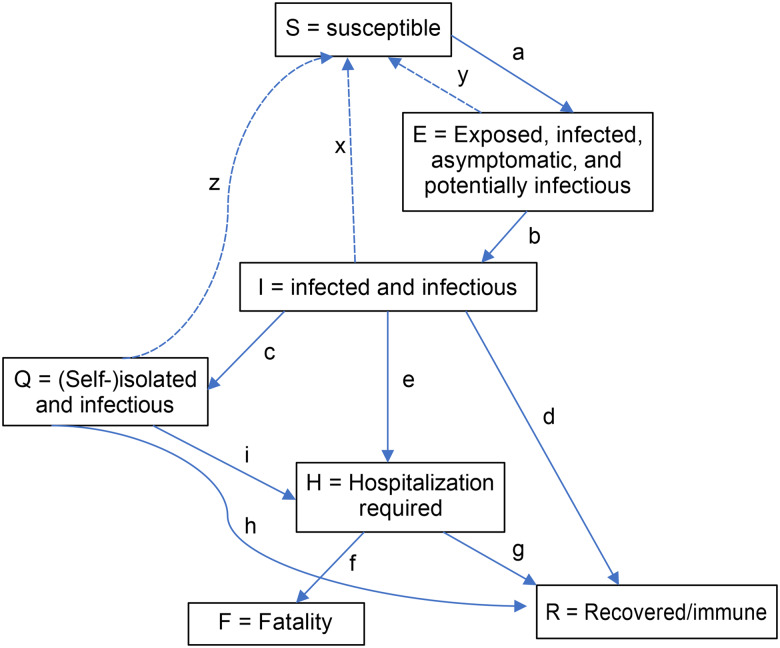
Flow diagram of the SEIQHRF model compartments and transition parameters (Appendix A: Table 1 provides additional information on the definitions and sources of the indicated transition parameters). SEIQHRF, susceptible, exposed, infected, quarantined and self-isolated, requiring hospitalisation, recovered/immune individuals, fatalities due to COVID-19.

The compartments and assumed transition parameters that drive the movement of individuals between these compartments are shown in Figure 2 [17]. Table 1 highlights the parameter symbols in the model flow diagram (Fig. 2) and provides a description of what they represent.

**Table 1.** Model parameters description

Diagram ref	Parameter	Parameter value	Description and source
x	act.rate.i	8	The number of exposure events (acts) between infectious individuals in the I compartment and susceptible individuals in the S compartment, per day [18]
x	inf.prob.i	0.05	Probability of passing on infection at each exposure event for interactions between infectious people in the I compartment and susceptible in S [19]
y	act.rate.e	8	The number of exposure events (acts) between infectious individuals in the E compartment and susceptible individuals in the S compartment, per day [18]
y	inf.prob.e	0.02	Probability of passing on infection at each exposure event for interactions between infectious people in the E compartment and susceptible in S. The rate is lower than inf.prob.i, reflecting the reduced infectivity of infected but asymptomatic people (~half of inf.prob.i) [15]
z	act.rate.q	2	The number of exposure events (acts) between infectious individuals in the Q compartment (isolated, self or otherwise) and susceptible individuals in the S compartment, per day. The rate is lower than for the I and E compartments, reflecting the much greater degree of social isolation for someone in (self-isolation) [15]
z	inf.prob.q	0.02	Probability of passing on infection at each exposure event for interactions between infectious people in the Q compartment and susceptible in S. The rate is lower than inf.prob.i, reflecting the greater care that self-isolated individuals will, on average, take regarding hygiene measures, such as wearing masks, to limit spread to others (~half of inf.prob.i) [15]
c	quar.rate	1/30	Rate per day at which symptomatic (or tested positive), infected I compartment people enter self-isolation (Q compartment). Asymptomatic E compartment people cannot enter self-isolation because they do not yet know they are infected. Default is a low rate reflecting low community awareness or compliance with self-isolation requirements or practices [15]
e,i	hosp.rate	1/100	Rate per day at which symptomatic (or tested positive), infected I compartment people or self-isolated Q compartment people enter the state of requiring hospital care – that is, become serious cases. A default rate of 1% per day with an average illness duration of about 10 days means a bit less than 10% of cases will require hospitalisation [15]
g	disch.rate	1/14	Rate per day at which people needing hospitalisation recover [20]
b	prog.rate	1/10	Rate per day at which people who are infected but asymptomatic (E compartment) progress to becoming symptomatic (or test-positive), the I compartment [15]
b	prog.dist.scale	5.1	Scale parameter for Weibull distribution for progression [21]
b	prog.dist.shape	1.5	Shape parameter for Weibull distribution for progression [21]
d	rec.rate	1/21	Rate per day at which people who are infected and symptomatic (I compartment) recover, thus entering the R compartment [22]
d	rec.dist.scale	35	Scale parameter for Weibull distribution for recovery [15]
d	rec.dist.shape	1.5	Shape parameter for Weibull distribution for recovery [15]
f	fat.rate.base	1/50	Baseline mortality rate per day for people needing hospitalisation (deaths due to the virus) [15]
f	hosp.cap	27	Number of available hospital beds per 1000 population in Saudi Arabia [23]
f	fat.rate.overcap	1/25	Mortality rate per day for people needing hospitalisation but who cannot get into hospital due to the hospitals being full. The default rate is twice that for those who do get into hospital [15]
f	fat.tcoeff	0.5	Time co-efficient for increasing mortality rate as time in the H compartment increases for each individual in it [15]
	a.rate	(18/365)/1000	Background demographic arrival rate – approximately the daily birth rate for Saudi Arabia [24]
	ds.rate, de.rate, de.rate, dq.rate, dh.rate, dr.rate	ds.rate, de.rate, de.rate, dq.rate, dh.rate, dr.rate = (3.5/365)/1000, dh.rate = (22/365)/1000	Background demographic departure (death not due to a virus) rates. Defaults based on Saudi Arabia crude death rates [25, 26]

### Model parameters

The model transition parameters were based largely on data from studies published during the COVID-19 pandemic. Demographic data for Saudi Arabia were obtained from various sources including websites of the official Ministry of Health and World Health Organization. Among all the parameters, some are a single rate (e.g. crude death rate), while others are rates drawn from probability distributions such as binomial or Weibull distributions. In instances where a parameter was randomly drawn from a probability distribution, the parameter values corresponding to the specific distribution are indicated. For example, the rate per day at which people who are infected but asymptomatic (E compartment) progress to becoming symptomatic (or test-positive) follows a Weibull distribution with a scale parameter for progression corresponding to 5.1 and shape parameter corresponding to 1.5. Therefore, the parameter values described in Table 1 are either used as rates or drawn from specific probability distributions.

The background mortality rate (deaths not due to the severe acute respiratory syndrome coronavirus-2 (SARS-CoV-2)) applies to all compartments [17]. The R0 was calculated assuming a serial interval with a mean *γ* distribution of 4.5 and a standard deviation of 3.4.

### Model assumptions

The model had the following assumptions: (1) exposed, infected, asymptomatic people are infectious; (2) the H compartment represents those needing hospitalisation, i.e. if hospitals had the capacity, they would be admitted; and (3) case fatalities are assumed to only occur in the H compartment regardless of whether the patients are hospitalised or not [17].

### Comparison of the intervention scenarios

We assessed the impact of a subset of NPIs that were implemented in Saudi Arabia and compared the following scenarios:

- Unmitigated scenario (baseline) – no action is taken except for self-isolation at a very low rate (1/30). This default rate reflects low community awareness or compliance with self-isolation requirements or practices, assuming that individuals are not mandated to self-isolate.
- Mitigation scenario – a 25% reduction in social contact compared to the baseline value through social distancing and a twofold increase in self-isolation rates, relative to the baseline scenario.
- Enhanced mitigation scenario – a 50% reduction in social contact compared to baseline values through social distancing and a twofold increase in self-isolation rates, relative to the baseline scenario.

In this model, we assumed that the reduction in social contact and increase in self-isolation rates were implemented gradually from day 15 (measured from the start of the epidemic) until day 45 and maintained at the maximum value thereafter. For instance, in the enhanced mitigation scenario, the contact rates were gradually scaled down from an average of eight contacts per day on day 15 to four contacts per day on day 45 and maintained constant thereafter. The impact of these interventions on the reduction in the numbers of new cases, deaths and individuals requiring hospitalisation was assessed.

The respective R0 values for the different scenarios were evaluated. They were computed for the growth phase of the epidemic, using the distribution of serial intervals (time between the onset of a primary case and time of onset in the secondary cases) based on maximum likelihood estimation.

### Model implementation

The SEIQHRF model was implemented using R statistical programming (The R Project for Statistical Computing. https://www.R-project.org/). Twenty simulation runs were undertaken and estimates from each were averaged to obtain the final estimates. Considering the computational intensity of running the simulations, parallel processing was employed using four computer processing units, with the model having a runtime of 365 days. We extracted the distributions of the timing of transitions to various compartments. This was done as a check to confirm that they were reasonable for the transition parameters that they represented and by defining a function that extracted the timing from the simulation results object. Then, we plotted the timing for visualisation. The modelling results were compared to those of the actual trajectory of the epidemic in Saudi Arabia.

## Results

### Compartment duration frequency distributions

**Fig. 3.**
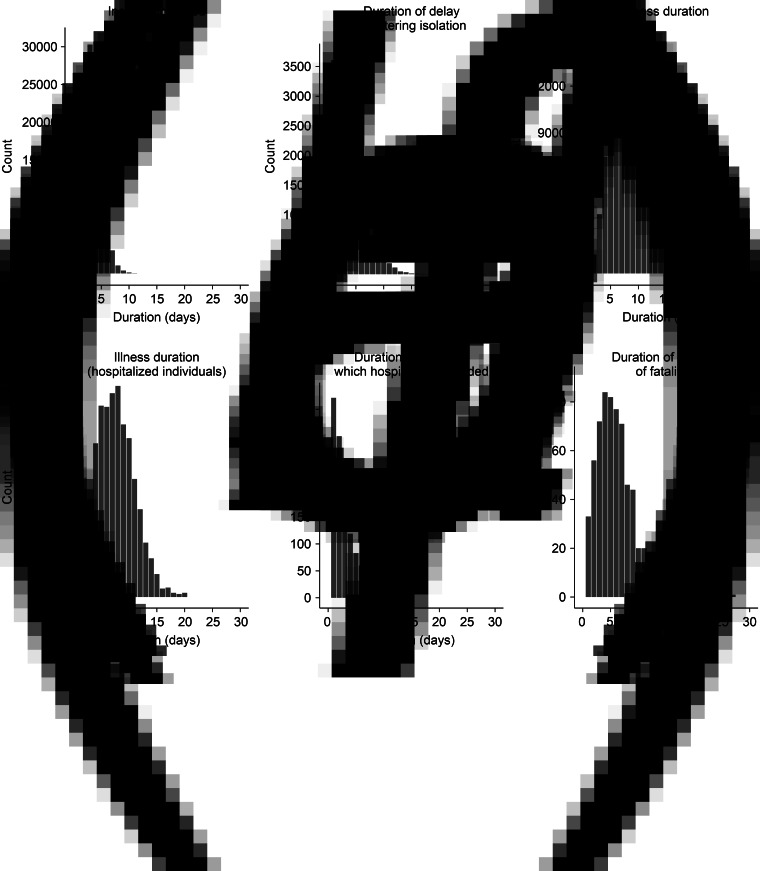
Distributions of the durations that individuals spent in each of the key compartments of the model. (a) Distribution of the incubation period, i.e. the period between the exposure to severe acute respiratory syndrome coronavirus 2 (SARS-CoV-2) and the onset of symptoms; (b) distribution of duration at which symptomatic (or tested positive), infected and infectious people enter self-isolation; (c) distribution of duration at which people who are infected and symptomatic recover; (d) distribution of duration at which people needing hospitalisation or are hospitalised recover; (e) distribution of duration at which symptomatic (or tested positive), infected and infectious people or self-isolated people enter the state of requiring hospital care; (f) distribution of survival duration of fatalities.

The distributions of the simulated durations spent in the key compartments of our model under the baseline scenario were found to be reasonable within the context of the existing literature (Fig. 3).

### Comparison of non-pharmacological intervention scenarios

#### Infected and asymptomatic individuals

**Fig. 4.**
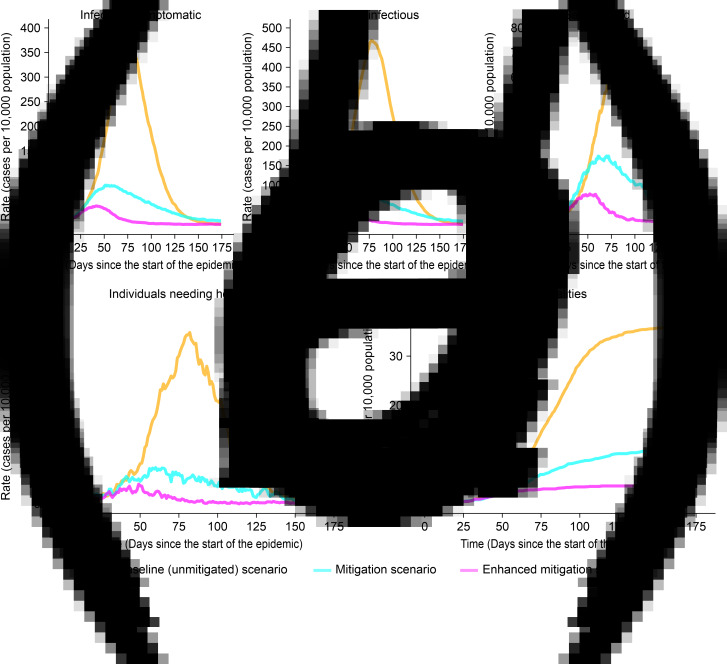
Trajectory of rates (cases per 10 000) per compartment for the baseline scenario *vs.* the two non-pharmaceutical intervention scenarios with variable levels of reduced social contact. The mitigation scenario assumes a 25% reduction in social contact through social distancing and a twofold increase in self-isolation rates; the enhanced mitigation scenario assumes a 50% reduction in social contact through social distancing and a twofold increase in self-isolation rates.

Compared to the unmitigated scenario, the mitigation and enhanced mitigation scenarios were found to reduce the peak number of infected but asymptomatic individuals by threefold and eightfold, from 382 to 81 and 382 to 46 per 10 000 population, respectively (Table 2; Fig. 4(a)). The infected and infectious cases peaked on days 73, 56 and 39 for the unmitigated, mitigation and enhanced mitigation scenarios, respectively.

**Table 2.** Comparison of baseline *vs.* NPI scenarios

Indicator	Scenario	Peak day	Number of people during the peak day
Infected and asymptomatic individuals	Unmitigated	73	382 per 10 000
	Mitigated	56	81 per 10 000
	Enhanced mitigation	39	46 per 10 000
Infected and infectious individuals	Unmitigated	82	472 per 10 000
	Mitigated	58	87 per 10 000
	Enhanced mitigation	42	52 per 10 000
Incident cases	Unmitigated	71	77 per 10 000
	Mitigated	54	16 per 10 000
	Enhanced mitigation	35	9 per 10 000
	**Observed**	**136**	**1.14 per 10 000**
Individuals requiring hospitalisation	Unmitigated	79	17 per 10 000
	Mitigated	61	4 per 10 000
	Enhanced mitigation	44	2 per 10 000
Fatality rate	Unmitigated	79	17 per 10 000
	Mitigated	61	4 per 10 000
	Enhanced mitigation	44	2 per 10 000
	**Observed**	**155**	**0.016 per 10 000**
R_0_ during the growth phase of the epidemic	Unmitigated		1.15
	Mitigated		1.14
	Enhanced mitigation		1.22
	**Observed**		**2.5**

NPI, non-pharmacological interventions.

#### Infected and infectious individuals

Compared to the unmitigated scenario, the mitigation and enhanced mitigation scenarios were found to reduce the peak number of infected and infectious individuals by fivefold and ninefold, from 472 to 87 and 472 to 52 per 10 000 population, respectively (Table 2; Fig. 4(b) and (c)). The infected and infectious cases peaked on days 82, 58 and 42 for the unmitigated, mitigation and enhanced mitigation scenarios, respectively.

#### Incident cases in modelling scenarios and the actual trajectory of the epidemic

**Fig. 5.**
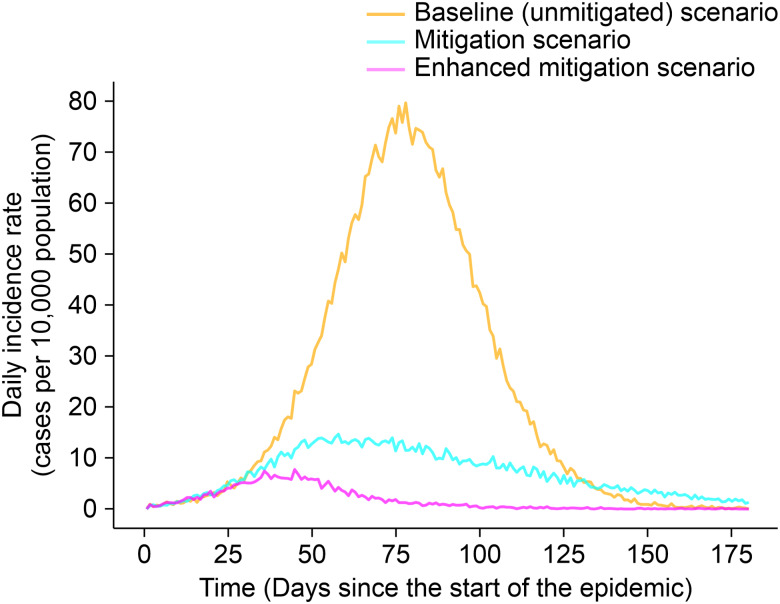
Incidence rates (cases per 10 000) for the baseline (unmitigated) scenario *vs.* the two non-pharmaceutical intervention scenarios with variable levels of reduced social contact. The mitigation scenario assumed a 25% reduction in social contact through social distancing and a twofold increase in self-isolation rates; the enhanced mitigation scenario assumed a 50% reduction in social contact through social distancing and a twofold increase in self-isolation rates.

Compared to the unmitigated scenario, the mitigation and enhanced mitigation scenarios reduced the daily incident cases fivefold and ninefold, from 77 to 16 and 77 to 9 per 10 000 population, respectively. The predicted peaks of the pandemic for the unmitigated, mitigation and enhanced mitigation scenarios were on days 71, 54 and 35, respectively, and the R0 were 1.15, 1.14 and 1.22, respectively. Conversely, the actual observed epidemic had a delayed peak and a lower incidence rate of 1.14 per 10 000 population on day 136 (Figs 1 and 5; Table 2).

#### Hospitalisation rate

**Fig. 6.**
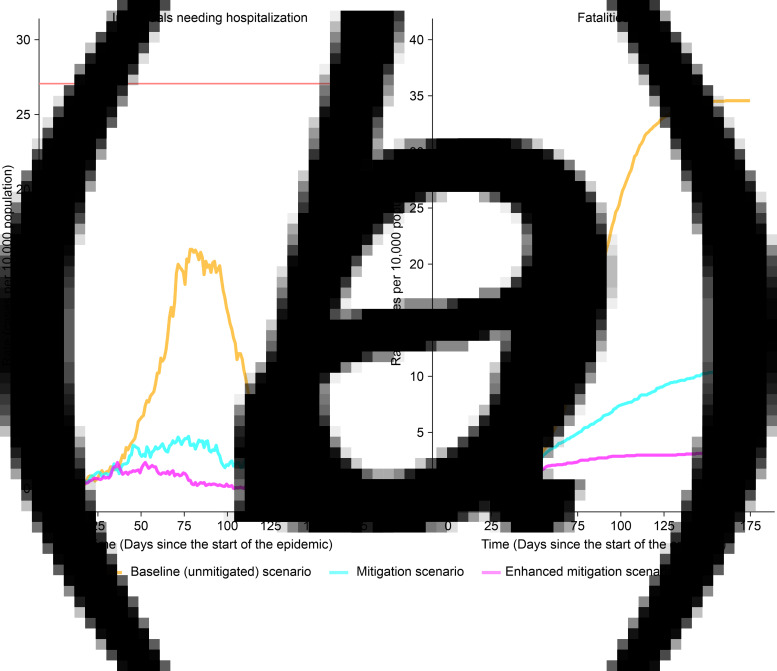
(a) Hospitalisation and (b) fatality rates (cases per 10 000) for the baseline (unmitigated) scenario *vs.* the two non-pharmaceutical intervention scenarios with variable levels of reduced social contact. The mitigation scenario assumed a 25% reduction in social contact through social distancing and a twofold increase in self-isolation rates; the enhanced mitigation scenario assumed a 50% reduction in social contact through social distancing and a twofold increase in self-isolation rates. The red line in (a) corresponds to the number of available hospital beds in the KSA (27 per 10 000).

The peak daily hospitalisation rates for the unmitigated, mitigation and enhanced mitigation scenarios were 17, 4 and 2 per 10 000 population, respectively, peaking on days 79, 61 and 44, respectively (Table 2; Figs 4(a) and 6(a)). With an average of 27 hospital beds per 1000 population in Saudi Arabia [27], the number of individuals requiring hospitalisation did not surpass the available hospital beds in any of the three scenarios (Table 2; Fig. 6(a)).

#### Fatality rate in modelling scenarios and the actual trajectory of the epidemic

Compared with the unmitigated scenario, the mitigation and enhanced mitigation scenarios reduced the highest fatality rate by threefold and sevenfold, from 35 to 13 and 35 to 5 per 10 000 population, respectively (Table 2; Figs 4(e) and 6(b)), and the fatalities peaked on days 150, 125 and 60, respectively (Table 2; Figs 4(e) and 6(b)). Conversely, the actual observed epidemic had a delayed peak and a lower fatality rate of 0.016 per 10 000 population on day 155.

#### Basic reproductive rate

The basic reproductive rates for the unmitigated, mitigated and enhanced mitigated scenarios were 1.15, 1.14 and 1.22, respectively. The R0 for the actual observed epidemic was 2.5 (Table 2). A possible explanation for the higher R0 in the actual observed trajectory than that in the enhanced mitigation scenario is that the actual trajectory shortened the pandemic, and thus, would have led to a higher R0 at the growth phase of the pandemic.

## Discussion

We aimed to assess the potential effect of NPI on the COVID-19 trajectory in Saudi Arabia using stochastic modelling and compare the results to the actual trajectory of the epidemic. Although the mitigation and enhanced mitigation scenarios had an earlier peak in the epidemic compared to the actual observed trajectory of the epidemic, both scenarios had considerably lower peaks. When comparing the incident cases and fatalities in the mitigation and enhanced mitigation scenarios to those of the actual observed epidemic, the incident cases and fatalities were considerably delayed, and their number was lower in the actual observed trajectory of the epidemic when compared to those in both mitigation scenarios. Conversely, the basic reproductive rate during the growth phase of the epidemic in the actual observed trajectory of the epidemic was higher than that in all three scenarios.

To the best of our knowledge, this is the first study that has examined the stochastic modelling of the COVID-19 pandemic and compared the results to the actual observed trajectory of the epidemic in the Kingdom of Saudi Arabia. However, there are some limitations. Stochastic models rely on estimates of proportions [28], and this has an impact on their accuracy and applicability. As COVID-19 was a new disease, most of the transmission parameters relied on early data available from China, some of which may not be applicable in Saudi Arabia, such as the estimated serial interval values. Furthermore, stochastic models assume that there is a heterogeneous mix of populations and that the susceptible population maintains a relatively constant size and structure. Moreover, stochastic models can be applied only to situations in which the number of infected people increases exponentially [8]. We also relied on contact rate estimates based on published data, which may have already changed in value by the start of the epidemic in Saudi Arabia. Additionally, we assumed that those who recovered from the disease gained immunity throughout the course of our epidemic projections; this may not be correct.

Both the mitigation and enhanced mitigation scenarios that had an earlier peak in the epidemic compared to that in the actual observed trajectory of the epidemic had considerably lower peaks when compared to the unmitigated scenario. In New York State and the USA, a social distancing regimen that reduced the contact rate by 10% from its baseline value was projected to reduce the number of daily hospitalisations and isolation of confirmed cases at the peak of the epidemic by 24% and 21%, respectively. Reducing the contact rate by 40% from its baseline value was projected to reduce the number of daily hospitalisations and isolation of confirmed cases at the peak of the epidemic by 92% and 88% and the number of deaths by 84% and 64% of the predicted baseline deaths in New York State and the USA, respectively [29]. In our model, which focused on social distancing and self-isolation strategies and assumed a decrease in the contact rates by 25% and 50% and twofold increase in self-isolation rates, there was a 76% and 88% reduction in the number of hospitalisations and 71% and 94% reduction in the number of fatalities in the mitigation and enhanced mitigation scenarios, respectively, when compared to those in the unmitigated scenario. In the Wuhan province of China, the NPIs that were implemented included strengthening case isolation, close contact tracing, cordoning off hotspots and traffic control. If no intervention had been taken, the number of cases would have been 51-fold higher than the actual number of cases [30]. Early case detection, contact isolation and inner-city contact reduction reduced the total number of cases by fivefold and two-and-a-half-fold, respectively. Limiting inter-city travel had no effect on the number of infections. Moreover, such measures were only effective when implemented in combination with early case detection, isolation and contact reduction [18]. These modelling results from Wuhan are comparable to that in the mitigation and enhanced mitigation scenarios in our model that focused on social distancing and an increase in self-isolation rate strategies and led to an approximately fivefold and ninefold incidence reduction, respectively, when compared to the unmitigated case scenario [19].

Conversely, peak incident cases and fatalities in the mitigation and enhanced mitigation scenarios were considerably earlier and higher when compared to that in the actual observed trajectory of the epidemic in Saudi Arabia. In Saudi Arabia, travel and entry restrictions were implemented on 2 February 2020, delaying the first reported case by 30 days [20]. Saudi Arabia imposed severe mobility restrictions and curfews in March and April 2020. These included the closure of educational institutions, malls, cafes, restaurants, and government offices and suspension of sports competitions and communal prayers from 8 March 2020 (Fig. 1) [21]. It is possible that these measures reduced the rate of social mixing within the population (more than that observed in the enhanced mitigation scenario [22]), and therefore, reduced the spread of infection [23] and resulting in the flattening of the curve and delaying of the peak. Additionally, with a high level of government trust, compliance with precautionary measures in Saudi Arabia may have also contributed to this effect [24]. In addition to the NPIs, Saudi Arabia was one of the first countries to test for SARS-CoV-2 using polymerase chain reaction. The testing focuses on the early identification of transmission chains, helps contain the epidemic and supports the institution of preventative measures to help reduce mortality and protect vulnerable people [25]. The algorithms put in place prioritised testing for those at highest risk, including health workers, immunocompromised individuals, the young and older adults, due to limited availability of test kits in early 2020. The rate of testing was increased using 24 h ‘Tetamman or “reassurance” Clinics’, followed by testing at drive-through centres and primary health care centres. By 4 July 2020, up to 53 000 tests were being conducted in the country daily [26]. By the end of August 2020, more than 5 million COVID-19 tests had been performed, corresponding to more than 15% of the Saudi population. At that time, Saudi Arabia was conducting 134 tests per 1000 people and 46.5 tests per confirmed case. The World Health Organization suggested that 10–30 tests per confirmed case was a benchmark for adequate testing and that a positivity rate of <5% was an indicator of the pandemic being under control. By May 2020, only 8–10% of persons tested for COVID-19 were testing positive in Jeddah, Saudi Arabia [31]. This implies that investing resources in testing was worthwhile.

Timely testing in a modelling study had the largest impact on reducing onward transmission [21]. Similar results have been observed using modelling in China where there were more than 2 million cases per month in the Hubei province alone before the lockdown. Following the closure of Wuhan on 23 January 2020, the potential number of cases in Hubei was predicted to decrease to 1 million per month [32]. The additional closure of the Hubei Province, 3 days later, was predicted to reduce the cumulative number of cases to 69 230 (R0 = 3.7). Following the initiation of mass screening in the Hubei Province on 12 February 2020, the cumulative number of cases was 66 386 (R0 = 3.4). Applying these measures on a longer-term basis would have led to a further reduction in the spread of the pandemic [18]. In Italy, Giordano *et al*. showed that a population-wide social distancing strategy combined with an effective testing strategy would considerably reduce the effect of the epidemic and help end it [13].

Conversely, the basic reproductive rate during the growth phase of the epidemic in the actual observed trajectory of the epidemic was higher than that in all three scenarios. The basic reproduction number (R0), defined as the average number of new cases caused by an infected individual in a susceptible population [32], is an indicator of viral transmissibility and can be used to estimate the number of cases in a population. When the R0 is >1, the number of cases typically increases [33]. In China, expanded testing and rapid availability of results contributed to early detection of cases, helping prevent further spread; the R0 reduced from 1.2 to 0.8. This is because there is a possible infectious period of 14 days during which it would be impossible to quarantine all close contacts [34]. In Italy, modelling showed that a partial lockdown reduced the R0 to 1.6, whereas a full-lockdown reduced it to 0.99. Widespread testing further reduced the R0 to 0.85. These results are comparable to those in the unmitigated and mitigation scenarios in our model wherein the R0 decreased from 1.15 to 1.14 [13]. Further reduction may not have been possible as our model did not include testing for COVID-19. The lower infection rates in the actual scenario with a high R0 could also be attributed to the reduction in social mixing. In Japan, using stochastic modelling, when the time spent in infectious zones was reduced, the number of infected individuals and spread of infection considerably decreased [10].

Although a higher R0 is associated with a lower probability of controlling the infection, the initial number of cases, time to isolation, transmission probability before the onset of symptoms, and proportion of asymptomatic cases lead to different probabilities of reducing an outbreak with R0 values of 1.5, 2.5 and 3.5 [35]. The basic reproductive rate is also affected by model assumptions and model structures as well as socio-behavioural, environmental and biological factors that affect pathogen transmission, and therefore, must be interpreted with caution [36].

## Conclusion

In conclusion, our modelling and its comparison with the actual observed trajectory of the COVID-19 epidemic in Saudi Arabia suggests that the unique and aggressive implementation of social distancing and self-isolation, marked reduction in social mixing, and mass testing contributed substantially to the currently observed downward trend of the disease spread, which otherwise would have had a far greater trajectory [37]. We recommend the use of extensive models that consider the natural history of the disease as well as social and behavioural patterns at the household and community levels, such as network-based stochastic simulation models, to fully elucidate the magnitude and trajectory of an epidemic. However, simple models, such as those used in this study, can be useful for informing the epidemic response while relying on age-specific data, actual network topology inferred from daily commute data or contact tracing, and a population factor in regions where the population is large or there is a high proportion of asymptomatic individuals [38]. Future studies should investigate the impact of different R0 values on the course of the epidemic with various public health interventions.

## Data Availability

The data that support the findings of this study are available from the corresponding author upon reasonable request.
